# A dataset on declared tax evasion attitudes of students and entrepreneurs from Poland under the slippery slope framework

**DOI:** 10.1016/j.dib.2023.109183

**Published:** 2023-04-25

**Authors:** Larissa M. Batrancea, Janusz Kudła, Barbara Błaszczak, Mateusz Kopyt

**Affiliations:** a Department of Business, Babes-Bolyai University, 7 Horea Street, Cluj-Napoca 400174, Romania; b Faculty of Economic Sciences, University of Warsaw, 44/50 Długa Street, Warsaw 00-241, Poland

**Keywords:** Taxation attitude, Tax shirking, Voluntary tax compliance, Enforced tax compliance, Ripple effect, Undertakers, Self-employed

## Abstract

The datasets included in this article come from a survey carried out on a group of Polish students and self-employed entrepreneurs and were originally created for studies on tax behaviour under the slippery slope framework. The slippery slope framework explains the role of extensive power execution and building trust in the tax administration in enhancing either enforced or voluntary tax compliance accordingly [1]. Students of economics, finance, and management at the Faculty of Economic Sciences and the Faculty of Management at the University of Warsaw were surveyed in two rounds, in 2011 and 2022, using paper-based questionnaires handed to them personally. Entrepreneurs were invited to fill in online questionnaires in 2020. Questionnaires were filled in by self-employed individuals from the Kuyavia-Pomerania, Lower Silesia, Lublin, and Silesia Province. The datasets provide 599 records for students and 422 observations for entrepreneurs. The purpose of collecting these data was to analyse the attitudes of the mentioned social groups towards tax compliance and tax evasion under the slippery slope framework along two dimensions: trust in authorities and power of authorities. The sample was selected because students in these fields are the most likely to become entrepreneurs, so the study sought to capture the potential behavioural change that is taking place. Each questionnaire consisted of three parts, a description of a fictitious country (Varosia) in one of four scenarios: (1) high trust-high power; (2) low trust-high power; (3) high trust-low power; (4) low trust-low power, 28 questions including manipulation checks on trust in authorities and power of authorities, intended tax compliance, voluntary tax compliance, enforced tax compliance, intended tax evasion, tax morale and the perceived similarity between Varosia and Poland, and finally two questions on gender and age of respondents. The data presented are particularly useful for policymakers in shaping tax policy and economists in analyses regarding taxation. Researchers may be interested in reusing the provided datasets for comparative research in other social groups, regions, and countries.

**Specifications Table**

**Table:** 

Subject	Economics, Econometrics, and Finance
Specific subject area	Taxation evasion attitude, taxation compliance attitude
Type of data	Table Questionnaire (PDF format with variable codes for data tables)
How the data were acquired	Paper-based questionnaires were used for students (in 2011 and 2022). The results were manually digitalised into CSV files. For self-employed entrepreneurs, online forms were used (2020) on a University LimeSurvey system. Email addresses (for sending invitations) were obtained from the Central Registration and Information on Business in Poland (CEIDG). Tokens were used to prevent multiple completion of the survey. The questionnaires were randomly distributed to the participants and were anonymous. Survey scenarios and items were adapted from Wahl et al. [2], Kirchler and Wahl [3] and Alm and Torgler [4]. The experimental design and questionnaires follow Kogler et al. [5] and Batrancea et al. [6].
Data format	Raw (Comma Separated Value - CSV and Stata ver. 16 - DTA files). There are two separate datasets, one for students and one for entrepreneurs. Each dataset is available in both of the above indicated formats and each format contains the same data.
Description of data collection	The voluntary survey was provided to students during classes of different fields and years of study. About 95% of the collected surveys were complete or almost complete. The self-employed from four Polish provinces filled in online survey with an opt-out option. 707 entrepreneurs responded, i.e. about 1.8% of the total number of invitations sent. More than half of the responses were fully completed. The datasets consist of 599 student and 422 entrepreneurs answers.
Data source location	• Data from students' survey:Institution: University of Warsaw (Faculty of Economic Sciences, Faculty of Management)City/Town/Region: Warsaw, Masovian ProvinceCountry: Poland • Data from entrepreneurs' survey:City/Town/Region: Kuyavia-Pomerania, Lower Silesia, Lublin, Silesia Province.Country: Poland
Data accessibility	Repository name: Mendeley Data Data identification number: 10.17632/96v79pw4gz.2 Direct URL to data: https://data.mendeley.com/datasets/96v79pw4gz
Related research article	L.M. Batrancea, J. Kudła, B. Błaszczak, M. Kopyt, Differences in tax evasion attitudes between students and entrepreneurs under the slippery slope framework, Journal of Economic Behavior & Organization, 200 (2022) 464-482. https://doi.org/10.1016/j.jebo.2022.06.017

## Value of the Data


 

• The value of the data is that it provides information from two independent samples of students and entrepreneurs, which can be used for comparative purposes when studying tax compliance attitudes. A researcher can determine whether the two groups differ significantly on a given factor and whether the student sample would be representative for analysing behavioural phenomena related to tax compliance.
• The datasets include 599 responses from students and 422 responses from entrepreneurs. The aim of the study was to analyse and compare the attitudes of these social groups towards their tax obligations. The main objective was to find out whether studies based on student samples, easier accessible to researchers, could be used as a proxy for real taxpayers in the analysis of tax compliance questionnaires.
• The collection of student data at two points in time also allowed comparisons to be made within groups. The datasets also include information on respondents' gender and age (as primary demographic variables), which extends the possibilities of analysis to include such aspects.
• The datasets are valuable for researchers and policymakers. The former can use insights from this study when conducting research in the fields of economics, political science, and psychology. The latter can use insights that could shape economic and political decisions, as the study uses scenarios that are close to real economic and political situations.
• The data presented can be used in comparative research by focusing on other social groups or by conducting cross-country comparisons. Our data can also be used to analyse perceptions of public authorities actions in the context of trust and power, taking into account the scenarios presented in the introductory section of the questionnaires.

## 1 Objective

The presented datasets were originally created for studies on tax behaviour under the slippery slope framework, which includes the dimensions of trust in authorities and power of authorities [1]. We investigated whether the slippery slope hypothesis is valid for the student population. It was also very helpful in an international comparison of tax evasion attitudes [6]. The original 2011 dataset on students was extended with the results of an analogous survey conducted on self-employed entrepreneurs (2020) and a new group of students (2022). The enlarged sample could shed some light on the changes in attitudes during the transition from students (2011) to entrepreneurs (2020), as these two groups belong to the same generation. As the socio-political situation has changed between 2011 and 2020ies, it opens up the possibility to compare whether students have changed their behaviour between 2011 and 2022. The data from the two groups of students (from 2011 and 2022) provides an opportunity to control for political and economic changes that may have affected the respondents’ attitudes. This has not been studied before. This data article can inspire further comparative studies with the related research paper, increasing its impact.

## 2 Data Description

The datasets in the repository [7] are provided in two formats: as raw data files with comma-separated value (CSV) and in a Stata ver. 16 (dta) format. In addition, the PDF file of the survey structure, that was the basis for data acquisition, is included. The *Entrepreneursvarosia* files (both in csv and dta format) contain data from the 2020 entrepreneurs survey, while the *Studentsvarosia* files (also in both formats) contain responses from the student surveys run in 2011 and 2022. The first dataset includes responses from a sample of 422 entrepreneurs, while the second dataset from a sample of 599 students (combined from both survey editions, observations starting with the number 10336 represent data collected in 2022). The first group of student observations (from 2011) includes 335 records, while the second group (from 2022) has 264 answers. The PDF file with the survey structure (*Survey.pdf*) presents all four scenarios used, according to the experimental design described in detail in the next section. The survey file also contains (in italics and square brackets) the names of the variables used in the raw datasets.

All raw data files have almost the same structure. The difference in the files describing the student responses (compared to the files with the entrepreneur responses) is only due to the specificity of the student data, which includes two research subgroups. An additional dummy variable [Sample_subgroup] has been included in the *Studentsvarosia* files to describe the survey year for the student group. A value of 0 indicates responses collected in 2011, while a value of 1 responses gathered in 2022. The variable [ID] is only included for technical reasons and it indicates the number of each questionnaire when it was digitalised. It can be considered as a raw label. In both datasets, the variables indicating the type of manipulation, namely, trust in authorities [Trust_sc] and power of authorities [Power_sc], were dummy coded with 1 = “low” and 2 = “high” to indicate four types of applied scenarios.

The answers to all 28 questions from the main part of the survey were based on Likert-type scale, with scores ranging from 1 to 9 (scale mean = 5). Details of the questions, possible answers, and variable codes are available in the attached PDF file in the repository [7]. In the case of three questions ‒ Intended tax compliance (question 3), Manipulation check trust (question 2), and Manipulation check power (question 2) ‒ they were structured so that the logic of their answers is opposite compared to the other two questions in each of their sections. Such a design allows for a better check of the answer reliability with the chosen statistical methods (e.g. Cronbach alpha). For these questions, however, the coding of participants' responses should be reversed. Therefore, both the original respondents' answers and the additional variables obtained through reverse coding have been included in the corresponding data files. The reverse coding results were calculated by subtracting the original value of the response to a given question from the value of 10. The values of the variables thus obtained are included in the columns labelled as follows: [Intended_TC_3_rev], [Check_Trust_2_rev], and [Check_Power_2_rev].

The datasets also include basic demographic information, i.e. the gender and age of the respondents. These variables are labelled [Age] and [Gender], respectively. Gender is dummy coded with 0 indicating “male” and 1 “female”. Age values are given as integers.

It should be noted that missing responses (missing data indicated as “NA” in csv files or empty fields in dta files) in individual answers occur only in the student dataset.

Tables 1,2 and 3 provide a basic summary of the contents of the datasets with some descriptive statistics for the variables collected during the study.

**Table 1:** Summary information on the number of responses in the student and entrepreneur datasets by survey scenario.

	Students 2011 Sample_subgroup=0	Students 2022 Sample_subgroup=1	Entrepreneurs 2020
Scenario 1 high trust (2)/high power (2)	84	64	106
Scenario 2 low trust (1)/high power (2)	83	69	81
Scenario 3 high trust (2)/low power (1)	84	70	116
Scenario 4 low trust (1)/low power (1)	84	61	119
**Total**	**335**	**264**	**422**

**Table 2:** Selected descriptive statistics of individual variables for student responses.

Variable	Students 2011 Sample_subgroup=0					Students 2022 Sample_subgroup=1				
	Obs.	Mean	Std. Dev.	Min.	Max.	Obs.	Mean	Std. Dev.	Min.	Max.
Intended_TC_1	334	6.66	2.46	1	9	264	7.02	2.33	1	9
Intended_TC_2	334	6.95	2.14	1	9	264	7.29	1.91	1	9
Intended_TC_3	332	4.05	2.74	1	9	264	3.89	2.78	1	9
Intended_TC_3_rev	332	5.95	2.74	1	9	264	6.11	2.78	1	9
Check_Trust_1	335	4.66	2.75	1	9	264	4.55	2.98	1	9
Check_Trust_2	332	5.52	2.57	1	9	264	5.49	2.82	1	9
Check_Trust_2_rev	332	4.48	2.57	1	9	264	4.51	2.82	1	9
Check_Trust_3	335	4.42	2.52	1	9	264	4.47	2.82	1	9
Check_Power_1	335	4.97	3.15	1	9	263	5.01	3.28	1	9
Check_Power_2	335	4.89	3.04	1	9	264	4.83	3.07	1	9
Check_Power_2_rev	335	5.11	3.04	1	9	264	5.17	3.07	1	9
Check_Power_3	335	4.68	3.00	1	9	264	4.73	3.12	1	9
Voluntary_TC_1	335	6.42	2.42	1	9	264	6.93	2.19	1	9
Voluntary_TC_2	334	5.32	2.43	1	9	264	5.84	2.34	1	9
Voluntary_TC_3	334	4.89	2.34	1	9	264	5.59	2.32	1	9
Voluntary_TC_4	335	5.95	2.44	1	9	263	6.53	2.25	1	9
Voluntary_TC_5	334	6.21	2.30	1	9	264	6.64	2.29	1	9
Enforced_TC_1	334	5.22	2.88	1	9	263	5.35	3.14	1	9
Enforced_TC_2	334	5.13	2.87	1	9	264	5.22	3.11	1	9
Enforced_TC_3	335	5.24	2.82	1	9	264	5.40	2.99	1	9
Enforced_TC_4	335	5.40	3.02	1	9	264	5.50	3.15	1	9
Enforced_TC_5	333	4.83	2.52	1	9	264	5.05	2.75	1	9
Tax_Evasion_1	335	5.10	2.62	1	9	264	5.02	2.75	1	9
Tax_Evasion_2	335	4.83	2.60	1	9	264	4.60	2.59	1	9
Tax_Evasion_3	335	5.43	2.62	1	9	264	5.12	2.65	1	9
Tax_Evasion_4	335	5.41	2.53	1	9	264	5.12	2.69	1	9
Tax_Evasion_5	334	4.89	2.50	1	9	264	4.21	2.55	1	9
Similarity_1	335	4.32	2.23	1	9	264	4.27	2.28	1	9
Similarity_2	333	4.11	2.29	1	9	264	4.18	2.33	1	9
Similarity_3	334	4.57	2.58	1	9	264	4.63	2.78	1	9
Tax_Morale	334	4.22	1.80	1	9	264	4.07	1.84	1	9
Age	334	22.29	1.89	18	35	262	21.45	1.92	18	35
Gender (0-male; 1-female)	335	0.58	0.49	0	1	260	0.65	0.48	0	1

**Table 3:** Selected descriptive statistics of individual variables for entrepreneur responses.

Variable	Entrepreneurs 2020				
	Obs.	Mean	Std. Dev.	Min.	Max.
Intended_TC_1	422	7.83	2.00	1	9
Intended_TC_2	422	7.80	1.87	1	9
Intended_TC_3	422	3.04	2.82	1	9
Intended_TC_3_rev	422	6.96	2.82	1	9
Check_Trust_1	422	4.70	3.03	1	9
Check_Trust_2	422	5.46	3.02	1	9
Check_Trust_2_rev	422	4.54	3.02	1	9
Check_Trust_3	422	4.41	2.94	1	9
Check_Power_1	422	4.95	3.15	1	9
Check_Power_2	422	4.78	3.11	1	9
Check_Power_2_rev	422	5.22	3.11	1	9
Check_Power_3	422	4.77	3.07	1	9
Voluntary_TC_1	422	7.56	2.19	1	9
Voluntary_TC_2	422	6.16	2.63	1	9
Voluntary_TC_3	422	5.70	2.75	1	9
Voluntary_TC_4	422	6.84	2.56	1	9
Voluntary_TC_5	422	6.92	2.48	1	9
Enforced_TC_1	422	4.44	3.02	1	9
Enforced_TC_2	422	4.32	3.08	1	9
Enforced_TC_3	422	4.47	3.02	1	9
Enforced_TC_4	422	4.83	3.34	1	9
Enforced_TC_5	422	3.70	2.75	1	9
Tax_Evasion_1	422	3.89	2.82	1	9
Tax_Evasion_2	422	3.78	2.84	1	9
Tax_Evasion_3	422	3.73	2.85	1	9
Tax_Evasion_4	422	4.03	2.92	1	9
Tax_Evasion_5	422	3.37	2.64	1	9
Similarity_1	422	4.75	2.72	1	9
Similarity_2	422	4.64	2.90	1	9
Similarity_3	422	4.77	2.94	1	9
Tax_Morale	422	3.39	1.99	1	9
Age	422	40.50	12.53	20	78
Gender (0-male; 1-female)	422	0.31	0.46	0	1

## 3 Experimental Design, Material and Methods

Each questionnaire used in our study consisted of three parts: 1) a description of a fictitious country Varosia presented in one of four variants (described below); 2) 28 questions concerning manipulation checks on trust in authorities and power of authorities, intended tax compliance, voluntary tax compliance, enforced tax compliance, intended tax evasion, opinion on the similarity between Varosia and Poland, and a question on tax morale; 3) demographic items on the gender and age of the respondent.

The four variants of the scenarios are each based on a manipulation of trust in and power of authorities within Varosia. The questionnaire begins with a vignette presenting the trust- and power-related features of Varosia. The variants are as follows: (1) high trust-high power; (2) low trust-high power; (3) high trust-low power; (4) low trust-low power. The high-trust scenarios portrayed tax authorities as highly respected by citizens, providing high-quality services to taxpayers and supporting them. The high-power scenarios characterised the authorities as efficiently identifying and sanctioning tax evaders based on a substantial budget available and skilled staff. The low-trust scenario describes authorities that are not trusted by citizens, provide low-quality public services, and do not support taxpayers. Similarly, the low-power scenario referred to ineffective prevention of tax evasion and low levels of tax enforcement. The questionnaires were randomly distributed to the participants (each participant received only one type of survey with the random scenario out of the four mentioned above). All respondents were informed that the questionnaires were anonymous. There was no remuneration (in any form) for taking part in the survey.

In the case of the student sample – both for 2011 and 2022 – the survey was provided during classes in a paper form. Participation in the survey was voluntary. Classes with a high number of potential attending students and from different fields of study and years of study were selected. The survey was carried out in two faculties specialising in the teaching of economics, management, and finance (the Faculty of Economic Sciences and the Faculty of Management at the University of Warsaw) among undergraduate and masters students, as they may reveal stronger or weaker attitudes towards tax evasion in their future lives (this is the case of students studying in the above-mentioned faculties). The questionnaires were collected in the first part of the semester of an academic year in order to increase the possible attendance of students (student participation in classes often decreases during the semester). The differentiation of student groups was also taken into account to avoid the possibility of the same student completing the questionnaire twice, e.g. the survey was only carried out once for a given year, programme and faculty. Students were given some time (several minutes) to prepare their answers and they could (or could not) return the questionnaires to the person conducting the research. The 2022 study was conducted after the full reopening of the universities in Poland (following the COVID-2019 pandemic lockdown). In 2022, the majority of the classes were held in person. The collected responses were manually entered into CSV files by a member of the research team. The other member of the research team checked for errors or omissions in the dataset to confirm that the process of CSV file preparation was correct.

Self-employed entrepreneurs were invited to fill in online questionnaires (in 2020). They received an email invitation to complete the survey. Their email addresses were obtained from the publicly available database of the Central Registration and Information on Business in Poland (CEIDG). The surveys were available on a dedicated LimeSurvey system installed on a server at the Faculty of Economic Sciences, University of Warsaw. Links to the survey from email invitations contained tokens preventing the same invitee from completing the survey multiple times. Despite the individual tokens in the online invitations, the LimeSurvey mechanism ensured the anonymity of responses. The sample contained information about the self-employed entrepreneurs from four Polish provinces: Kuyavia-Pomerania, Lower Silesia, Lublin, and Silesia Province. We decided to use data from economically differentiated provinces in order to better reflect possible differences in the perception of entrepreneurship in different social and economic environments. Lower Silesia is the most developed province (after the capital district which is dominated by large multinational companies), Lublin is the least developed province in Poland, Silesia is highly developed but with problems of restructuring heavy industry (coal mining), while Kuyavia-Pomerania is of the average affluence. The 2020 entrepreneur surveys were conducted before the introduction of the COVID-2019 pandemic lockdown in Poland (the lockdown started in March 2020).

It should be noted that both the content of the scenarios and the questions regarding the experimental manipulation, voluntary tax compliance, enforced tax compliance, intended tax compliance, and intended tax evasion were taken from the studies by Wahl et al. [2] and Kirchler and Wahl [3]. Furthermore, this experimental design was also used in the studies by Kogler et al. [5] and Batrancea et al. [6]. The tax morale variable was taken from Alm and Torgler [4].

## Ethics Statements

Local regulations do not require ethical approval from the relevant local ethics committee for surveys used to collect data for this publication (based on the Regulations of the Faculty of Economic Sciences Ethics Commission). All survey participants were given the opportunity to voluntarily opt out of completing the questionnaire. Participants were also informed that the survey was anonymous and that the confidentiality of their personal data (if applicable) would be strictly maintained.

## CRediT authorship contribution statement

**Larissa M. Batrancea:** Conceptualization, Methodology, Supervision, Writing – review & editing. **Janusz Kudła:** Conceptualization, Methodology, Supervision, Data curation, Investigation, Writing – review & editing. **Barbara Błaszczak:** Visualization, Investigation, Writing – original draft. **Mateusz Kopyt:** Software, Investigation, Data curation, Writing – original draft, Writing – review & editing.

## Declaration of Competing Interest

The authors declare that they have no known competing financial interests or personal relationships that could have appeared to influence the work reported in this paper.

## Data Availability

A dataset on declared tax evasion attitudes of students and entrepreneurs from Poland under the slippery slope framework (Original data) (Mendeley Data).
